# Exploring the In Vitro Antidiabetic Potential of Metal Oxide Nanoparticles Synthesized Using Lemongrass and Mint Formulation

**DOI:** 10.7759/cureus.53489

**Published:** 2024-02-03

**Authors:** Rajeshkumar Shanmugam, Tharani Munusamy, Afrin Nisha M, Annika Rajaselin, Sulochana Govindharaj

**Affiliations:** 1 Nanobiomedicine Lab, Centre for Global Health Research, Saveetha Medical College and Hospital, Saveetha Institute of Medical and Technical Sciences, Saveetha University, Chennai, IND; 2 Pharmacology, Saveetha Dental College and Hospitals, Saveetha Institute of Medical and Technical Sciences, Saveetha University, Chennai, IND

**Keywords:** green synthesis, zinc oxide nanoparticles, copper oxide nanoparticles, anti-diabetic activity, sustainable nanoparticles, product development, biomedical application

## Abstract

Aim

This study aimed to compare the antidiabetic effect of metal oxide nanoparticles (CuONPs and ZnONPs) prepared using lemongrass and mint herbal formulations.

Introduction

The study explores green-synthesized nanoparticles for potential applications in diabetes management, emphasizing sustainable synthesis methods, particularly zinc oxide nanoparticles (ZnONPs) and copper oxide nanoparticles (CuONPs) produced from lemongrass and mint herbal formulations. The study was prompted by the increasing importance of innovative therapeutic strategies, responding to emerging health challenges, and leveraging advancements in nanotechnology and eco-friendly practices to explore the potential of green-synthesized nanoparticles in diabetes management.

Methods

The methods involve herbal formulation preparation, CuONPs and ZnONPs synthesis, and UV-visible spectrophotometry for characterization. In vitro antidiabetic activity is assessed through α-amylase and β-glucosidase enzyme assays using varied nanoparticle concentrations (10-50 µL).

Results

Visual observations confirm successful synthesis, with distinct color changes observed in both CuONPs and ZnONPs after 24 hours. UV-visible spectrophotometry reveals absorption peaks at 440 nm and 380 nm for CuONPs and ZnONPs, respectively. In the α-amylase assay, both nanoparticles exhibit concentration-dependent inhibition, with CuONPs ranging from 40% to 77% and ZnONPs ranging from 36% to 80%. The β-glucosidase assay demonstrates similar concentration-dependent inhibition patterns, highlighting significant differences.

Conclusion

The study concludes that CuONPs and ZnONPs synthesis using lemongrass and mint herbal formulations show concentration-dependent antidiabetic activity. The comparative analysis underscores the need for tailored approaches based on nanoparticle composition. These findings contribute valuable insights into the therapeutic potential of green-synthesized nanoparticles, paving the way for future nanomedicine research and development in diabetes management.

## Introduction

In recent years, metal oxide nanoparticles have emerged as promising candidates in the field of biomedical materials, owing to their unique physicochemical properties and versatile applications. Among these, zinc oxide nanoparticles (ZnONPs) and copper oxide nanoparticles (CuONPs) have garnered considerable attention due to their distinctive characteristics that render them suitable for various biomedical applications such as drug delivery, biosensing, tissue therapy, immunotherapy, diagnosis, and regenerative medicine [1-3]. ZnONPs, synthesized using different plant extracts, have demonstrated in vitro antioxidant and antidiabetic activities, showcasing their potential for therapeutic applications [4].

Notably, the green synthesis of ZnONPs using plant extracts such as *Azadirachta indica, Hibiscus rosa-sinensis, Murraya koenigii, Moringa oleifera, and Tamarindus indica* has revealed antioxidant properties through free radical scavenging assays [5]. Moreover, ZnONPs synthesized using *Tamarindus indica* extract displayed remarkable antioxidant and antidiabetic activities [6]. While the focus on ZnONPs has elucidated their potential, CuONPs synthesized with plant extracts have also demonstrated promising antidiabetic activities in vitro. The inhibition of α-amylase and α-glucosidase, crucial enzymes in glucose metabolism, by CuONPs synthesized with various plant extracts presents a compelling avenue for diabetes management [7]. Lemongrass and mint, well-known for their diverse beneficial properties, have been extensively studied for their antimicrobial, anti-inflammatory, and antioxidant activities [8,9]. Lemongrass, rich in phenolic metabolites, terpenoids, and alkaloids, has been recognized for its antifungal, antibacterial, and anti-inflammatory properties [10]. On the other hand, mint is renowned for its cooling and soothing effects, making it a popular choice in oral care products and aromatherapy [11,12].

In this study, the synthesis of ZnONPs and CuONPs was carried out using a formulation incorporating lemongrass and mint extracts. This novel approach aims to harness the synergistic antidiabetic properties of both metal oxide nanoparticles and plant extracts, with a particular focus on α-amylase and β-glucosidase inhibition. The exploration of such combinations holds significant potential for advancing therapeutic strategies in diabetes management. This research contributes to the evolving landscape of nanomedicine by integrating the unique properties of metal oxide nanoparticles with the diverse bioactive compounds present in lemongrass and mint extracts, opening avenues for innovative and effective antidiabetic interventions.

## Materials and methods

Preparation of lemongrass and mint herbal formulation

One gram of dried lemongrass powder and one gram of mint powder were measured and added to 100 mL of distilled water. This aqueous formulation was kept on a heating mantle at 60-70 degrees Celsius for 15-20 minutes. After that, it was extracted using Whatman No. 1 filter paper, and this filtered herbal extract was stored in the refrigerator at 4 degrees Celsius for further synthesis of nanoparticles.

Green synthesis of CuONPs

A total of 30 mM of copper sulfate was dissolved in 50 mL distilled water to make a precursor solution. To the precursor solution, 50 mL of filtered herbal formulation was added. This mixture was kept in a magnetic stirrer at 600 rpm for 24-48 hours. The nanoparticle synthesis was analyzed using a UV-visible double-beam spectrophotometer at specific time intervals. After this, the nanoparticle solution was subjected to centrifugation at 8000 rpm for 10 minutes. The centrifugation process is done to separate the CuONP pellet from the supernatant. The collected pellet was stored in an airtight Eppendorf tube and the supernatant was discarded.

Green synthesis of ZnONPs

A total of 30 mM of zinc nitrate was dissolved in 50 mL distilled water to make a precursor solution. To the precursor solution, 50 mL of filtered herbal formulation was added. This reaction mixture was kept in a magnetic stirrer at 600 rpm for 24-48 hours. The nanoparticle synthesis was analyzed using a UV-visible double-beam spectrophotometer at specific time intervals. After this, the nanoparticle solution was subjected to centrifugation at 8000 rpm for 10 minutes. The centrifugation process is done to separate the ZnONP pellet from the supernatant. The collected pellet was stored in an airtight Eppendorf tube and the supernatant was discarded.

Comparative in vitro antidiabetic activity

In the in vitro antidiabetic assay, two techniques were employed: α-amylase inhibitory assay and β-glucosidase enzyme inhibitory assay.

α-Amylase Assay

To assess α-amylase inhibition, the liberation of maltose was examined in the α-amylase inhibitory assay. The procedure adhered to a protocol adapted from earlier research [7]. CuONPs and ZnONPs at concentrations ranging from 10 to 50 µg/mL were pre-incubated with 100 µg/mL of α-amylase solution at 37 degrees Celsius for 30 minutes. A starch solution containing 100 µg/mL (1% w/v) was added, and the mixture was incubated at 37 degrees Celsius for an additional 30 minutes. Subsequently, 3,5-dinitrosalicylic acid solution (DNSA) at a concentration of 96 mM was added. The reaction was halted, and the solution was subjected to. Sodium phosphate buffer (pH 6.9) was either used as a control or in an equivalent quantity to the enzyme extract. The absorbance was measured at 540 nm using an enzyme-linked immunosorbent assay (ELISA) plate reader. The experiment was conducted in triplicate, and acarbose served as the positive control. The percentage of α-amylase inhibition was calculated as follows:

% of inhibition = C-T/C x 100, where C is control and T is test sample.

β-Glucosidase Assay

In the β-glucosidase enzyme inhibitory assay, CuONPs and ZnONPs at concentrations of 10 to 50 µg/mL were mixed and a starch solution (2% w/v maltose or sucrose) was added in the presence of 0.2 M Tris buffer at pH 8.0. The mixture was incubated at 37 degrees Celsius for 5 minutes. Subsequently, 1 mL of β-glucosidase enzyme (1 U/mL) was added, and the reaction was allowed to proceed at room temperature for 40 minutes. The reaction was terminated with the addition of 2 mL of 6 N HCl. Acarbose served as the positive control. The absorbance was measured at 540 nm using an ELISA plate reader and the percentage of β-glucosidase inhibition was determined by the formula:

% of inhibition = C-T/C x 100, where C is control and T is test sample.

Statistical analysis

In this study, all experiments were conducted in triplicate to ensure the reliability of the results. The antidiabetic activity was measured, and the data were subjected to statistical analysis using standard error (SE). The standard error provides an estimate of the variability of the sample means, allowing for the assessment of the precision of the results.

## Results

Visual observation

**Figure 1:**
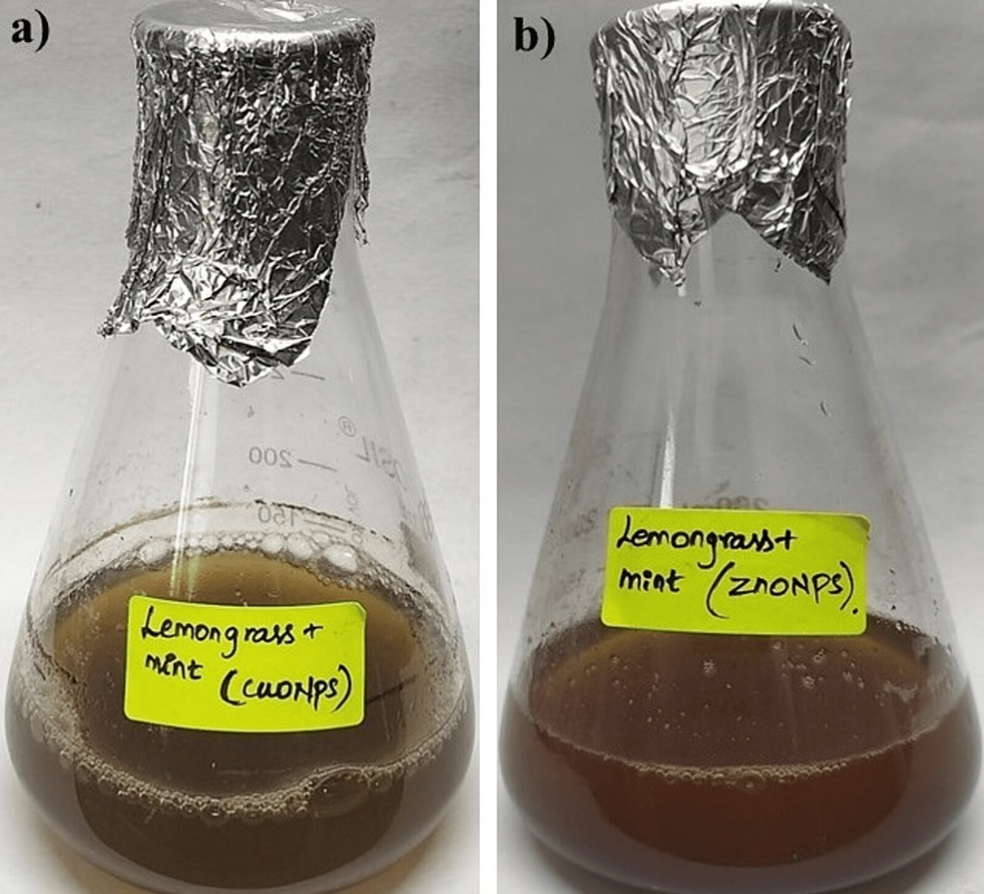
Visual observation of green-synthesized metal oxide nanoparticles: (a) Copper oxide nanoparticles using lemongrass and mint; (b) Zinc oxide nanoparticles using lemongrass and mint CuONPs: Copper oxide nanoparticles; ZnONPs: Zinc oxide nanoparticles

In Figure 1, the lemongrass and mint herbal formulations were employed for the synthesis of CuONPs and ZnONPs, and their visual characteristics were examined. After 24 hours, a significant color change was observed in both nanoparticles. For CuONPs, the initial bluish-green tint transformed into a distinct greenish-brown shade, indicating a successful alteration in the nanoparticles' physicochemical properties. On the other hand, ZnONPs exhibited a noticeable shift from a pale red color to a deep brown tone within the same time frame. These distinct color changes not only signify the completion of the synthesis process but also suggest the potential efficacy of lemongrass and mint herbal extracts as effective reducing and stabilizing agents for the green synthesis of CuONPs and ZnONPs.

UV-visible spectroscopy

**Figure 2:**
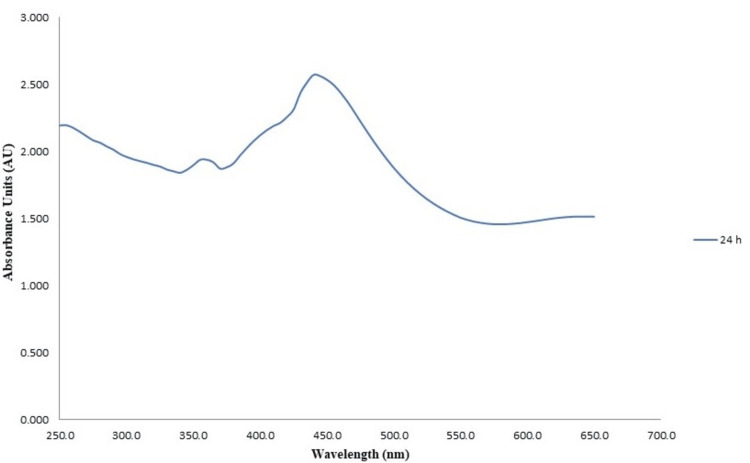
UV-visible spectra of green-synthesized copper oxide nanoparticles UV-visible spectra analysis of copper oxide nanoparticles synthesized through green methods using lemongrass and mint formulation. The measurements were taken 24 hours post-synthesis to assess the optical properties of the nanoparticles.

**Figure 3:**
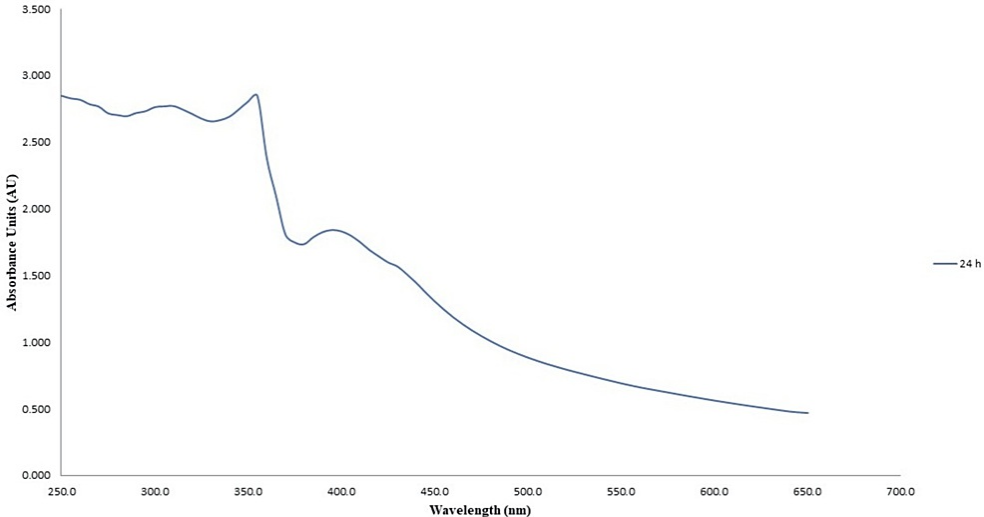
UV-visible spectra of green-synthesized zinc oxide nanoparticles UV-visible spectra illustrating the optical characteristics of zinc oxide nanoparticles synthesized through eco-friendly methods employing lemongrass and mint extracts. The measurements were recorded 24 hours post-synthesis to analyze the optical properties of the green-synthesized zinc oxide nanoparticles.

The results of the UV-visible spectrophotometry analysis for the green synthesis of CuONPs revealed a prominent absorption peak at 440 nm after 24 hours of incubation time as shown in Figure 2. Conversely, the analysis for ZnONPs exhibited a maximum absorption peak at 380 nm following 24 hours of incubation time as shown in Figure 3. These distinct absorption peaks signify the characteristic wavelengths at which these nanoparticles displayed their strongest absorbance, indicating their unique optical properties and confirming successful synthesis at the specified time intervals.

Antidiabetic activity of ZnONPs

**Figure 4:**
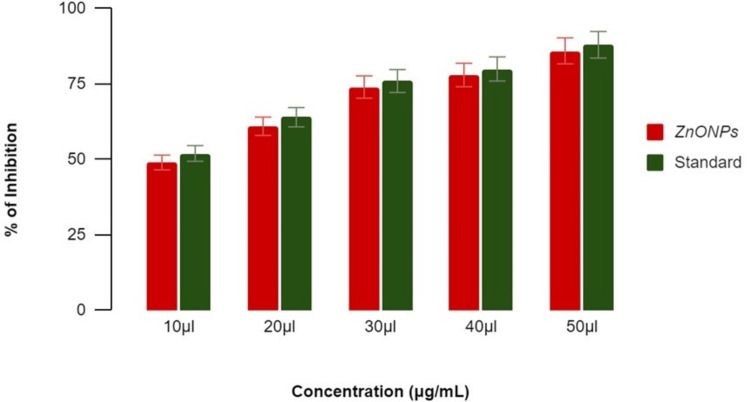
Antidiabetic activity of zinc oxide nanoparticles using α-amylase assay The term 'inhibition' refers to the reduction or decrease in enzymatic activity, while 'standard' refers to the benchmark (acarbose) used for comparative analysis. ZnONPs: Zinc oxide nanoparticles

**Figure 5:**
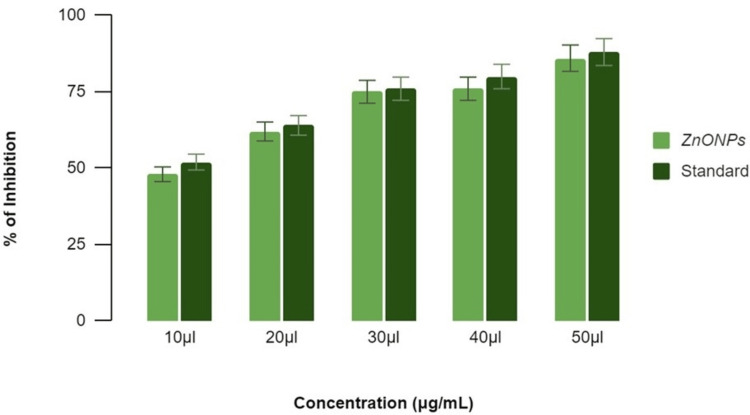
Antidiabetic activity of zinc oxide nanoparticles using β-glucosidase inhibitory assay The term 'inhibition' refers to the reduction or decrease in enzymatic activity, while 'standard' refers to the benchmark (acarbose) used for comparative analysis. ZnONPs: Zinc oxide nanoparticles

The investigation into the antidiabetic activity of ZnONPs, synthesized using lemongrass and mint herbal formulations, demonstrated significant inhibitory effects on α-amylase and β-glucosidase activities, as depicted in Figures 4-5. The concentration-dependent reduction in enzymatic activity suggests a potential mechanism for glycemic control. The ZnONPs-induced inhibition of α-amylase implies a diminished breakdown of complex carbohydrates, leading to reduced glucose production. Simultaneously, the observed inhibition of β-glucosidase points to a potential modulation of glucose absorption in the small intestine. The utilization of acarbose as the standard for comparative analysis provides insights into the potential of ZnONPs to modulate carbohydrate metabolism and glucose absorption. These combined effects propose a role for lemongrass and mint-synthesized ZnONPs in mitigating postprandial hyperglycemia, underscoring their promising application for further exploration in the realm of antidiabetic therapeutics.

α-Amylase Assay

Lemongrass and mint (ZnONPs) exhibited a concentration-dependent inhibition, with percentages of 36%, 50%, 62%, 74%, and 80% at concentrations of 10 μL, 20 μL, 30 μL, 40 μL, and 50 μL, respectively, as shown in Figure 4. In comparison, the standard acarbose showed percentages ranging from 47% to 84% at the corresponding concentrations. These results indicate the potential of ZnONPs in modulating α-amylase activity, with the herbal formulation influencing their inhibitory effects.

*β-Glucosidase* *Inhibitory Assay*

In the β-glucosidase enzyme assay, lemongrass and mint (ZnONPs) demonstrated concentration-dependent inhibition. At concentrations of 10 μL, 20 μL, 30 μL, 40 μL, and 50 μL, the inhibition percentages were 50%, 58%, 64%, 68%, and 78%, respectively, as shown in Figure 5. The standard reference displayed a similar concentration-dependent inhibitory pattern, with percentages ranging from 55% to 81%. These findings underscore the potential antidiabetic activity of ZnONPs, with the herbal formulation contributing to their observed inhibitory effects on β-glucosidase. Both α-amylase inhibitory activity and β-glucosidase inhibitory activity of ZnONPs synthesized using lemongrass and mint herbal formulation increased in a dose-dependent manner as compared with the standard drug acarbose.

Antidiabetic activity of CuONPs

**Figure 6:**
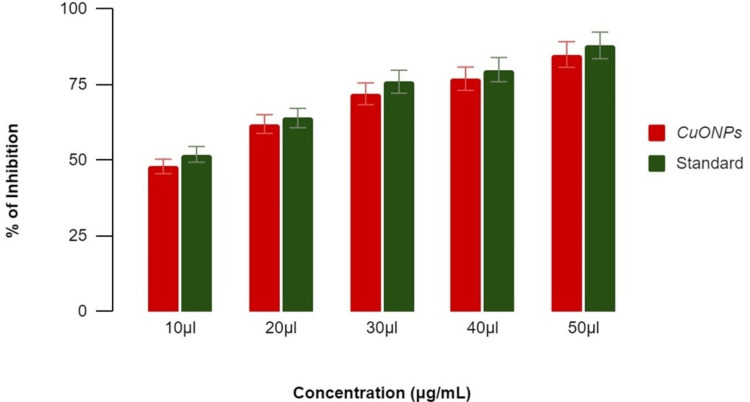
Antidiabetic activity of copper oxide nanoparticles using α-amylase inhibitory assay The term 'inhibition' refers to the reduction or decrease in enzymatic activity, while 'standard' refers to the benchmark (acarbose) used for comparative analysis. CuONPs: Copper oxide nanoparticles

**Figure 7:**
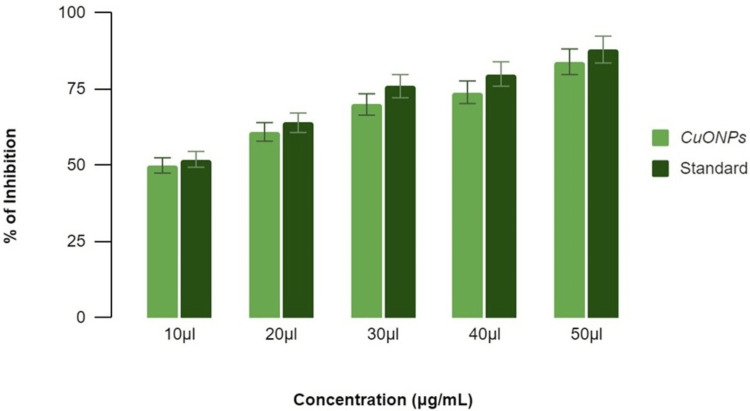
Antidiabetic activity of copper oxide nanoparticles using β-glucosidase inhibitory assay The term 'inhibition' refers to the reduction or decrease in enzymatic activity, while 'standard' refers to the benchmark (acarbose) used for comparative analysis. CuONPs: Copper oxide nanoparticles

Thein vitro antidiabetic activity of lemongrass and mint-synthesized CuONPs was rigorously evaluated through α-amylase and β-glucosidase enzyme assays, as presented in Figures 6-7. The utilization of acarbose as the standard for comparative analysis provides insights into the potential of CuONPs to modulate carbohydrate metabolism and glucose absorption. The subsequent sections detail these findings, shedding light on the promising antidiabetic attributes of lemongrass and mint-synthesized CuONPs.

α-Amylase Assay

CuONPs, derived from the lemongrass and mint herbal formulations, displayed a concentration-dependent inhibition of α-amylase activity. Specifically, at concentrations of 10 μL, 20 μL, 30 μL, 40 μL, and 50 μL, the inhibition percentages were 40%, 53%, 65%, 71%, and 77%, respectively, as shown in Figure 6. These results were compared against a standard reference, which exhibited comparable concentration-dependent inhibitory effects ranging from 47% to 84%. The observed inhibitory pattern underscores the potential of green-synthesized CuONPs to modulate α-amylase activity, a key enzyme associated with diabetes.

*β-Glucosidase In* *hibitory Assay*

In the β-glucosidase enzyme assay, CuONPs demonstrated concentration-dependent inhibition. At concentrations of 10 μL, 20 μL, 30 μL, 40 μL, and 50 μL, the corresponding inhibition percentages were 53%, 62%, 67%, 70%, and 79%, respectively, as shown in Figure 7. This concentration-specific inhibitory effect aligns with a similar pattern exhibited by the standard reference, which displayed percentages ranging from 55% to 81%. These detailed results not only highlight the concentration-specific efficacy of CuONPs but also emphasize the influence of the herbal formulations in enhancing the antidiabetic properties of these nanoparticles. The data suggest a potential avenue for the development of novel therapeutic strategies leveraging green-synthesized CuONPs in diabetes management.

## Discussion

The present study aimed to investigate and compare the potential antidiabetic properties of CuONPs and ZnONPs synthesized using a green approach mediated by lemongrass and mint herbal formulations. The research encompassed the synthesis process, characterization through UV-visible spectrophotometry, visual observation, and subsequent in vitro antidiabetic assays targeting α-amylase and β-glucosidase enzymes.

Previous studies have explored the in vitro antidiabetic efficacy of CuONPs and ZnONPs. Notably, biosynthesized CuONPs have demonstrated hypoglycemic effects in alloxan-induced diabetic rats [13]. Similarly, ZnONPs derived from *Tridax procumbens* leaf extract exhibited antidiabetic potential in streptozotocin-induced diabetic rats, effectively reducing blood glucose levels, preventing weight loss, and improving lipid profiles and glycated hemoglobin levels [14]. The literature also emphasizes the antidiabetic capabilities of ZnONPs, as well as other nanoparticles like selenium (Se) nanoparticles, magnesium oxide (MgO) nanoparticles, copper (Cu) nanoparticles, and cerium oxide (CeO2) nanoparticles [15]. Furthermore, ZnONP synthesis from *Areca catechu* leaf extract has shown antidiabetic properties in yeast models, suggesting their potential as an alternative diabetes treatment [16]. These findings collectively contribute to the body of evidence supporting the potential of various nanoparticles, including CuONPs and ZnONPs, as promising candidates for antidiabetic interventions. The successful green synthesis of CuONPs and ZnONPs was confirmed through UV-visible spectrophotometry, revealing distinct absorption peaks at 410 nm and 380 nm, respectively. Visual observations of color changes in both nanoparticles further indicated the completion of the synthesis process, highlighting the efficacy of lemongrass and mint herbal extracts as reducing and stabilizing agents [17,18].

Thein vitro antidiabetic activity of CuONPs and ZnONPs was assessed through α-amylase and β-glucosidase enzyme assays, which are crucial enzymes implicated in diabetes management. The concentration-dependent inhibition of α-amylase activity by CuONPs suggests their potential to modulate carbohydrate metabolism [19]. The percentage inhibitions at various concentrations (10 μL to 50 μL) ranged from 40% to 77%, showing a notable concentration-dependent pattern. Similarly, the β-glucosidase assay demonstrated concentration-dependent inhibition, with percentages ranging from 53% to 79%. These findings underscore the promising antidiabetic activity of CuONPs, indicating their ability to interfere with key enzymatic processes associated with diabetes. In the case of ZnONPs, the α-amylase assay revealed inhibition with percentages ranging from 36% to 80%. The β-glucosidase assay also demonstrated concentration-dependent inhibition, with percentages ranging from 50% to 78%. These results suggest that ZnONPs synthesized with lemongrass and mint herbal formulations possess significant antidiabetic potential, with observed inhibitory effects on α-amylase and β-glucosidase enzymes [20].

The comparative analysis between CuONPs and ZnONPs indicates distinct concentration-dependent inhibitory patterns in both α-amylase and β-glucosidase assays. While CuONPs showed slightly higher inhibition percentages in the α-amylase assay, ZnONPs exhibited comparable or higher inhibition percentages in the β-glucosidase assay. These variations could be attributed to the specific interactions between the nanoparticles and the enzymes, emphasizing the importance of nanoparticle composition in determining their biological effects. The intricate nature of these interactions implies further investigation to identify the underlying mechanisms influencing the differential inhibitory patterns observed.

Limitations

Despite providing valuable insights into the optical properties and antidiabetic potential of lemongrass and mint-synthesized CuONPs and ZnONPs, the study possesses some limitations. While the UV-visible spectrophotometry analysis confirmed successful synthesis with distinctive absorption peaks, the in vitro nature of the study restricts the direct applicability of these optical properties to the complex in vivo environment. Additionally, the promising antidiabetic attributes demonstrated by ZnONPs and CuONPs lack an exploration of nanoparticle stability and toxicity, essential for translational applications. The study's focus on short-term observations hinders a thorough understanding of sustained effects and stability. Addressing these limitations through in vivomodels, a comprehensive safety assessment, and longer-term observations will enhance the robustness of the findings and broaden their potential implications for antidiabetic therapeutics.

## Conclusions

This study revealed the significant antidiabetic potential of CuONPs and ZnONPs synthesized using lemongrass and mint herbal formulations. The observed concentration-dependent inhibitory effects on α-amylase and β-glucosidase enzymes highlight the therapeutic relevance of these nanoparticles in diabetes management. The comparative analysis distinctly portrays the behavior of CuONPs and ZnONPs, emphasizing the necessity for tailored approaches in harnessing their unique properties for specific therapeutic applications.These findings supports the exploration of green-synthesized nanoparticles as promising candidates for future antidiabetic drug development. The distinct characteristics of CuONPs and ZnONPs uncovered in this study pave the way for an effective and targeted therapeutic strategies in diabetes care.
